# Collagen Mimetic Peptides Promote Corneal Epithelial Cell Regeneration

**DOI:** 10.3389/fphar.2021.705623

**Published:** 2021-08-16

**Authors:** Robert O. Baratta, Brian J. Del Buono, Eric Schlumpf, Brian P. Ceresa, David J. Calkins

**Affiliations:** ^1^ Stuart Therapeutics, Stuart, FL, United States; ^2^ Department of Ophthalmology and Visual Sciences, University of Louisville School of Medicine, Louisville, KY, United States; ^3^ Department of Ophthalmology and Visual Sciences, Vanderbilt Eye Institute, Vanderbilt University Medical Center, Nashville, TN, United States

**Keywords:** corneal collagen damage, collagen mimetic peptides, ocular surface disease, collagen molecular activity, collagen reparative, anti-inflammation, regeneration

## Abstract

The cornea of the eye is at risk for injury through constant exposure to the extraocular environment. A highly collagenous structure, the cornea contains several different types distributed across multiple layers. The anterior-most layer contains non-keratinized epithelial cells that serve as a barrier to environmental, microbial, and other insults. Renewal and migration of basal epithelial cells from the limbus involve critical interactions between secreted basement membranes, composed primarily of type IV collagen, and underlying Bowman’s and stromal layers, which contain primarily type I collagen. This process is challenged in many diseases and conditions that insult the ocular surface and damage underlying collagen. We investigated the capacity of a collagen mimetic peptide (CMP), representing a fraction of a single strand of the damaged triple helix human type I collagen, to promote epithelial healing following an acute corneal wound. *In vitro*, the collagen mimetic peptide promoted the realignment of collagen damaged by enzymic digestion. In an *in vivo* mouse model, topical application of a CMP-containing formulation following a 360° lamellar keratectomy targeting the corneal epithelial layer accelerated wound closure during a 24 h period, compared to vehicle. We found that the CMP increased adherence of the basal epithelium to the underlying substrate and enhanced density of epithelial cells, while reducing variability in the regenerating layer. These results suggest that CMPs may represent a novel therapeutic to heal corneal tissue by repairing underlying collagen in conditions that damage the ocular surface.

## Introduction

Collagen plays a well-known role as a biomechanical structural support for the integrity of most tissues and organs. Collagens represent the major component of the extracellular matrix (ECM) and basement membranes, while in all connective tissues, including in the eye, collagens provide the major structural backbone. Disruption of and damage to the collagen in these structures inhibit recovery of tissues that they support.

In addition to providing biomechanical structural support, collagens serve a variety of additional functions. For example, specific cell-surface and intracellular receptors interact with collagens. Signaling by these receptors plays a key role in cellular adhesion, differentiation, growth, and other cellular activities, as well as the survival of cells both *in vivo* and *in vitro* (Vogel, 2001; Gelse et al., 2003). Collagens also are involved in the entrapment, local storage, and delivery of growth factors and cytokines. Through these receptor interactions and storage and delivery functions, collagen plays a key role in organ development, wound healing, and tissue repair (Hay, 1981; Yamaguchi et al., 1990; Zhu et al., 1999; Schlegel et al., 2004; Chattopadhyay and Raines, 2014; Kumar et al., 2014). Likewise, damage to collagen, particularly to the triple helix domain that is found in all collagen types, including type I collagen, disrupts this “cell interaction” role (San Antonio et al., 2020).

Collagen is a primary component of the cornea of the eye, contributing some 90% of the thickness through its distribution in distinct layers (Meek, 2009). The anterior-most corneal epithelium contains non-keratinized cells that protect against a variety of insults or injuries. The renewal and migration of basal epithelial cells occur at the limbus, which is the border between the transparent cornea and the opaque sclera of the eye (Mobaraki et al., 2019). This process includes secretion of type IV collagen-based basement membrane for adherence to underlying Bowman’s and stromal layers, which are primarily type I collagen, and is challenged in conditions that insult the ocular surface (reviewed in Baratta et al., 2021). Thus, the corneal epithelium and its basement membrane rely on the integrity of underlying collagen networks for immediate support and integrity.

The replacement cycle of collagen is quite slow in most tissues, including the cornea (Paik et al., 2018). We have suggested elsewhere that a tissue repair technology that could reestablish collagen fibrillar organization could restore both the biomechanical, structural role of collagen membranes and normal and healthy cell signaling (Baratta et al., 2021). A signature characteristic of collagen is a triple helical structure—a set of three polypeptide chains comprising repeating sequences of glycine-x-y triplets where x and y often (but not always) represent proline and hydroxyproline (Kadler et al., 2007). To this end, collagen mimetic peptides (CMPs) show great promise for their capacity to directly repair damaged triple helical collagen. CMPs are synthesized as fractional single strands of type I collagen and are specifically designed as fragments that intercalate into damaged and/or are partially digested triple helices, thus restoring their role in the structure and signaling (Chattopadhyay et al., 2012; Chattopadhyay et al., 2016; Chattopadhyay and Raines, 2014). For example, fragments of collagens IV, XV, and XVIII promote the growth of blood vessels and tumor cells and influence a variety of other cellular activities (Ortega and Werb, 2002). Synthesized CMPs of collagen type I specifically target areas of collagen disruption associated with skin wounds by reforming the native triple helix through intercalating into disrupted collagen (Chattopadhyay et al., 2012; Chattopadhyay et al., 2016).

Here, we test the capacity of a CMP delivered topically as an eyedrop to encourage rapid epithelial cell regrowth following an acute injury to the mouse cornea. We find that compared to a vehicle, CMP treatment accelerated the reestablishment of the epithelial layer and promoted structural adherence of the basal epithelium to the underlying anterior stroma surface. By reducing variability during regeneration, CMPs may show broad promise as therapeutics for ocular surface disease and injury.

## Materials and Methods

### Collagen Mimetic Peptides

We produced a single-strand, seven-repeat ((Pro-Pro-Gly)_7_) CMP (manufactured by Bachem, AG, Germany). This CMP does not form triple helices with itself but rather intercalates with high selectivity into damaged endogenous type I collagen as shown in both *in vitro* and *in vivo* experimental models (Chattopadhyay et al., 2012; Chattopadhyay et al., 2016; Chattopadhyay and Raines, 2014). The CMP was dissolved in phosphate buffered saline, which served as a vehicle, at concentrations of 25 and 250 nM. To demonstrate the effect of CMP treatment on collagen alignment, we coated Petri dishes with human type I collagen in sterile double distilled water at 100 μg/ml and incubated at 37°C for 2 h. These dishes were then treated with 100 U/ml collagenase at 37°C for 1.5 h, rinsed with phosphate buffered saline (PBS) to remove excess collagenase, and then exposed to either TNC buffer solution or CMP (100 µM) at 37°C for 12 h and again rinsed. Following Kivanany et al. (2018), we used differential interference contrast optics at ×60 magnification to obtain photomicrographs from multiple samples of each condition (*n* = 7), which were edge-enhanced using MATLAB (MathWorks, Natick, MA). The alignment of collagen strands was determined using MATLAB routines as previously described (Cooper et al., 2018).

### Animal Model of Corneal Epithelial Wounding

All animal experiments were in accordance with and approved by the University of Louisville Institutional Animal Care and Use Committee. Adult (8–10 weeks) female C57BL6/J mice from Jackson Laboratory (Bar Harbor, ME, United States) were maintained in 12 h light/dark cycle and allowed water and standard rodent chow ad libitum. Based on the variability measured in the previous use of this model of corneal wound healing, cohort size was set at *n* = 7 (Peterson et al., 2014; Rush et al., 2016). Following precedence in this field, we chose female mice due to their generally slower corneal healing rate (Wang et al., 2012). To investigate the ability of the CMP to promote healing of the ocular surface, we damaged the corneal epithelium using our previously published technique (Peterson et al., 2014; Rush et al., 2016). Mice were anesthetized with an intraperitoneal injection of ketamine (100 mg/kg) and xylazine (8 mg/kg; Butler Schein, Dublin, OH, United States). Under surgical microscopy control, we used a 1.5 mm diameter standard corneal surgical trephine centered on the mouse eye to demarcate a symmetric 360° circular incision in one cornea only of each mouse. This corresponds to a wound exposure covering about 70% of the corneal diameter limbus to the limbus (Henriksson et al., 2009). The wound was created manually using an Algerbrush ® II with a 0.5 mm burr (Alger Company, Inc., Lago Vista, TX, United States) by removing the epithelium and epithelial basement membranes through abrasive contact with the superficial (anterior) stroma. The wound bed was copiously irrigated with a balanced saline solution to remove remnants of epithelial cells. Immediately after wounding, corneas were visualized using sterile fluorescein sodium ophthalmic strips USP (Fluorets; Chauvin Laboratory, Aubenas, France) dampened with sterile PBS and obtained photomicrographs of all eyes using a stereoscopic zoom microscope (SMZ1000, Nikon, Tokyo, Japan) equipped with a digital sight DS-Fi2 camera (Nikon). After imaging, wounded eyes were treated with a single drop (10 μl) of either PBS or CMP (25 nM or 250 nM). Based on the published work of Peterson et al. (2014) and Rush et al. (2016), the interval between 16 and 24 h offers the greatest difference between untreated wounds and wounds treated with known pro-regenerative factors. Thus, we estimated effect size based on these earlier results and imaged the cornea in anesthetized mice as described above at 16 and 24 h post-injury. The area of each wound was measured using ImageJ software.

### Histology and Quantification

Following sacrifice at 24 h, enucleated eyes were placed in 4% paraformaldehyde overnight, washed, and embedded in paraffin for histological sectioning and hematoxylin and eosin (H&E) staining to visualize the regenerated corneal epithelium. From differential interference contrast micrographs, we quantified, using ImageJ, the density of stroma layers per unit area (in arbitrary units), length of junctions of complete adherence between regenerated basal epithelium and the underlying anterior stromal surface, the number of epithelial cell nuclei in the basal layer in random samples of fixed area across the cornea, and epithelial surface variability. The latter was determined by quantifying the number of nuclei in adjacent segments of a fixed area covering the wound zone and determining how the number in each segment deviated from the average for the sample. Sections (3–4/eye) were chosen randomly through a series containing the central-most 20–30% cornea by a naïve observer. From these, 30–100 micrographs were sampled from regions of interest, depending on the measurement, by a naïve observer. Quantification of outcomes as described was conducted blindly.

### Statistical Analysis

All data are presented as mean ± standard error of the mean (SEM). Statistical analyses and graphs were made using Sigma Plot Version 14 (Systat, San Jose, CA). Outlier analysis was performed using Grubbs’ test (Graphpad Software, San Diego CA). Parametric statistics were performed (*t*-test, analysis of variance) if data passed normality and equal variance tests; otherwise, we performed non-parametric statistics (Mann–Whitney, ANOVA on Ranks). Statistical significance was defined as *p* ≤ 0.05.

## Results

### Collagen Mimetic Peptides Accelerate Corneal Wound Healing

**FIGURE 1:**
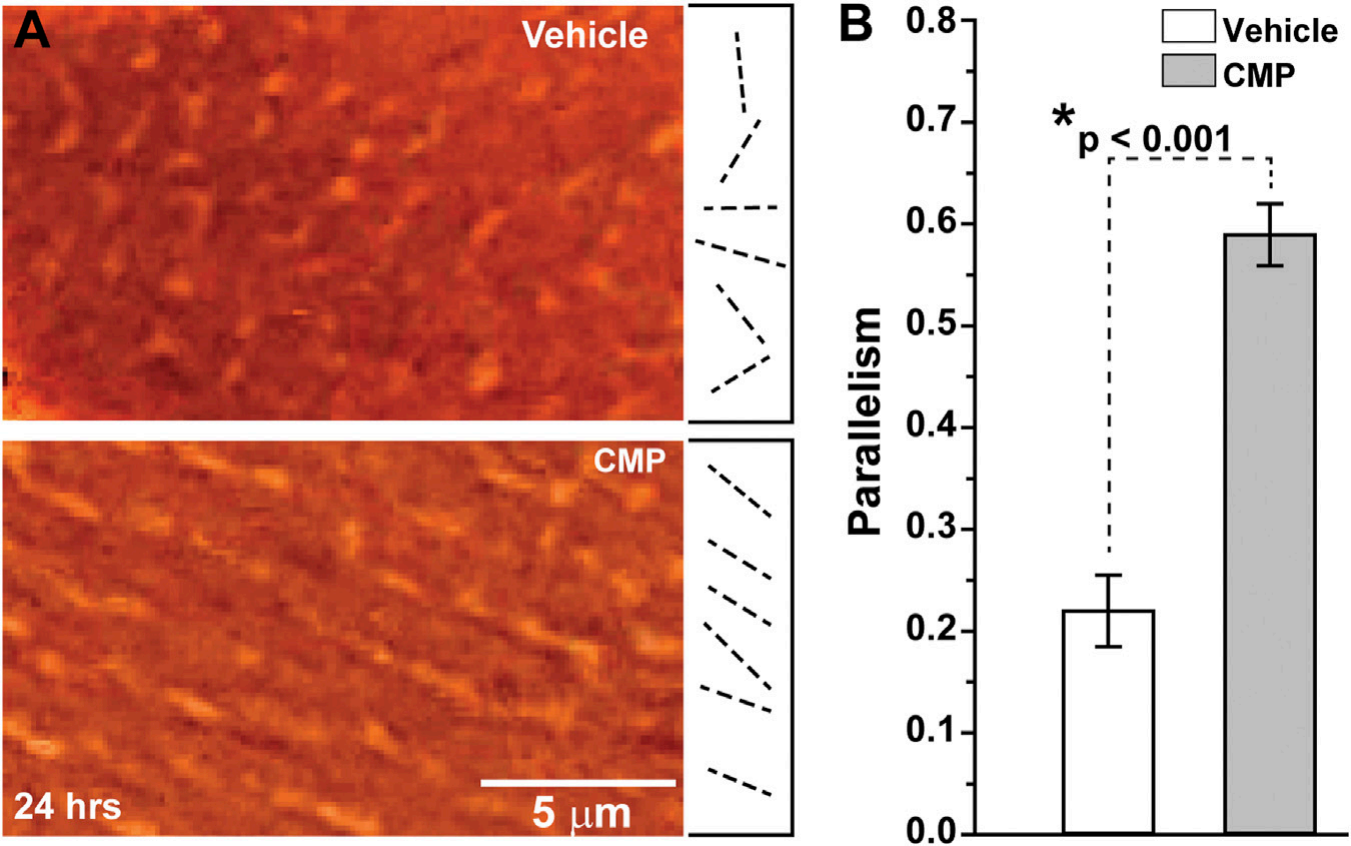
CMP realigns collagen fibers damaged by collagenase *in vitro.* **(A)** Edge-enhanced differential interference contrast images of applied type I collagen damaged by collagenase and treated with a vehicle (top) or CMP (bottom). Example vectors (dashed lines) assigned by algorithm show a higher degree of parallelism in CMP-treated preparations. **(B)** Vectors in preparations treated with CMP have a higher average tendency for parallel alignment compared to a vehicle (*: *p* < 0.001). Data: mean ± SEM; *n* = 7.

When damaged by collagenase, collagen that had been coated onto plates demonstrated highly disorientated strands, as shown by differential interference contrast microscopy (Figure 1A, top). Treatment with a CMP following collagenase treatment appeared to promote alignment of the collagen strands in parallel orientation (Figure 1A, bottom), similar to collagen in endogenous tissues in the absence of injury (Hapach et al., 2015). Parallel alignment is known to promote corneal keratocyte migration *in vitro* (Kivanany et al., 2018). We quantified the tendency for parallel alignment following an algorithm that assigns vectors to individual collagen strands in multiple random images sampled from the preparations (Cooper et al., 2018; Kivanany et al., 2018). This algorithm then calculates the overall tendency, in which a value of 1 indicates perfect parallelism and 0 corresponds to random alignment. Treatment of collagenase-damaged collagen with CMP improved alignment three-fold compared to a vehicle (*p* < 0.001; Figure 1B).

**FIGURE 2:**
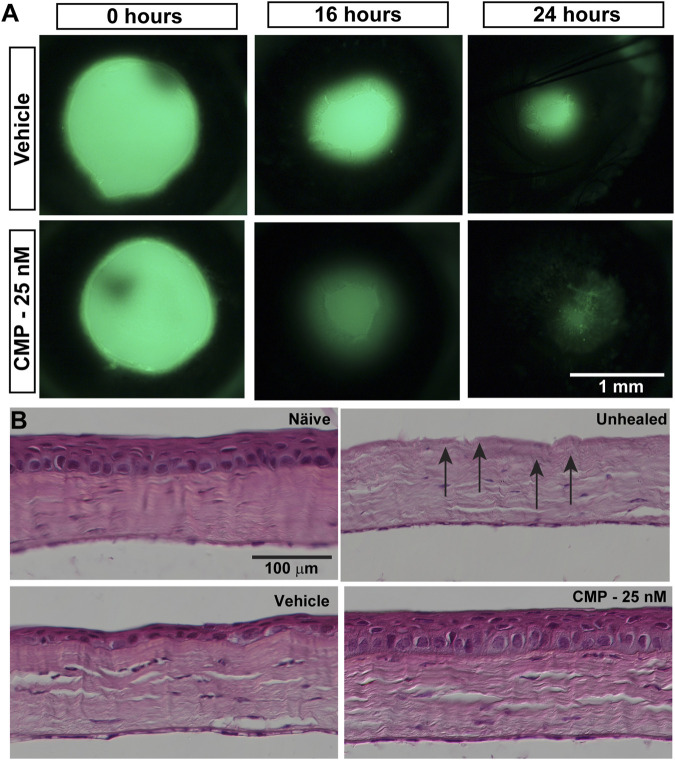
CMP promotes healing of corneal epithelium in mice. **(A)** Representative images showing residual corneal epithelial wounds visualized by fluorescein staining *in vivo* at the time of the initial wound (0 h) and 16 and 24 h later. Treatment with CMP (25 nM shown) appeared to accelerate the regeneration of the epithelium, as seen by smaller wound areas at 16 and 24 h. **(B)** Representative differential interference contrast images of H&E-stained histological sections through naïve and wounded cornea either left unhealed (by immediate sacrifice, arrows) or treated with either a vehicle or CMP and sacrificed 24 h later.

Given our finding that CMP treatment can rapidly repair collagen fibrils damaged by enzymic digestion *in vitro* and its demonstrated reparative capacity for cutaneous wounds (Chattopadhyay et al., 2016), we tested its influence on the repair of epithelial cells *in vivo* following an acute injury of the cornea in mice. After wounding, topical fluorescein defined the area of epithelial injury by penetrating the underlying exposed corneal stroma (Figure 2A). Initial wound size, determined by measuring this area, did not differ between vehicle- and CMP-treated eyes (*p* = 0.38). Compared to vehicle-treated eyes, treatment with CMP (25 nM) appeared to accelerate healing early. At both 16 and 24 h following wounding, the area of residual injury appeared smaller for eyes treated with CMP compared to those treated with a vehicle (Figure 2A). This was not due to differences in wound depth. In post-mortem histological sections (Figure 2B), the density of stroma for both vehicle- and CMP-treated eyes did not differ from either naïve stroma or stroma in eyes obtained immediately following wounding without a healing period (*p* = 0.15). This indicates consistency in the depth of the wound we created to remove the epithelium and its basement membrane.

**FIGURE 3:**
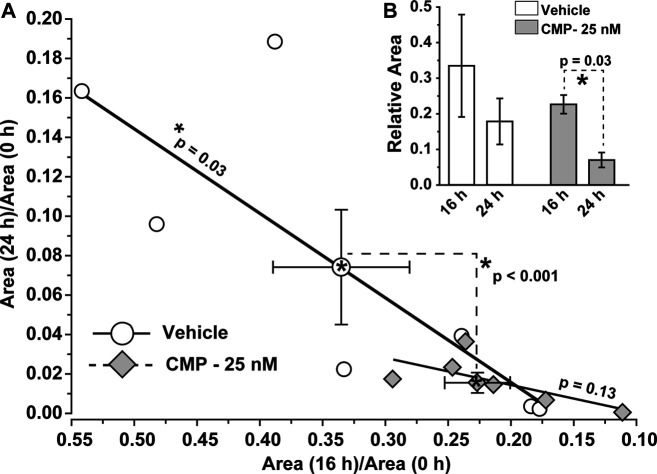
CMP accelerates the rate of epithelium wound closure. **(A)** For vehicle-treated eyes, the ratio of residual wound area at 24 h to initial (0 h) size decreases with a diminishing ratio of the area at 16 h to initial. The slope of the best-fitting regression line differs significantly from 0 (*, *p* = 0.03). For CMP-treated eyes (25 nM), accelerated closure clusters both ratios at smaller values, yielding an insignificant regression (*p* = 0.13). The cluster of ratios for a vehicle vs. CMP cohorts differed significantly (dashed lines, *p* < 0.001), as shown by multivariable analysis of variance to compare the means (mean ± SEM) (**B**, inset). Relative wound size calculated as the ratio of residual wound area at 16 h to initial and at 24 compared to 16 h for vehicle- and CMP-treated eyes (mean ± SEM). Wound area diminished significantly between 24 and 16 h compared to the initial 16 h period for CMP eyes (*p* = 0.03) but not for a vehicle (*p* = 0.10). Importantly, initial wound size for CMP-treated eyes (1.41 ± 0.13 mm) did not differ from vehicle-treated eyes (1.54 ± 0.17 mm, *p* = 0.38; *n* = 7 for each cohort).

To determine whether these qualitative trends were significant, we calculated how the ratio of residual wound size changed during the 24 h experimental period. For vehicle-treated eyes, the ratio of the residual wound area at 24 h to initial size (0 h) depended significantly on the ratio of wound size at 16 h to initial (*p* = 0.03; Figure 3A). For CMP-treated eyes, the ratio of residual to initial wound area clustered tightly at smaller values for both 16 and 24 h, with no dependence between the two (*p* = 0.13; Figure 3A). The difference between a vehicle and CMP clusters was significant (*p* < 0.001), as determined by multivariable analysis of variance with time post-injury and treatment condition as variables. Similarly, the rate of wound closure accelerated for CMP-treated eyes between 16 and 24 h. The ratio of residual size at 24 compared to 16 h was significantly smaller than 16 compared to initial wound size for CMP-treated eyes (*p* = 0.03), but not for the vehicle (Figure 3B). These results indicate that treatment with CMP accelerates corneal wound healing over time, beginning with smaller residual wounds by 16 h compared to initial wound size. Interestingly, wound closure for both 16 and 24 h was 15–20% slower for a higher concentration of CMP (250 nM) compared to the 25 nM concentration used in the studies shown in Figure 2 (data not shown).

### Collagen Mimetic Peptides Improve Reorganization of Regenerated Corneal Epithelium

**FIGURE 4:**
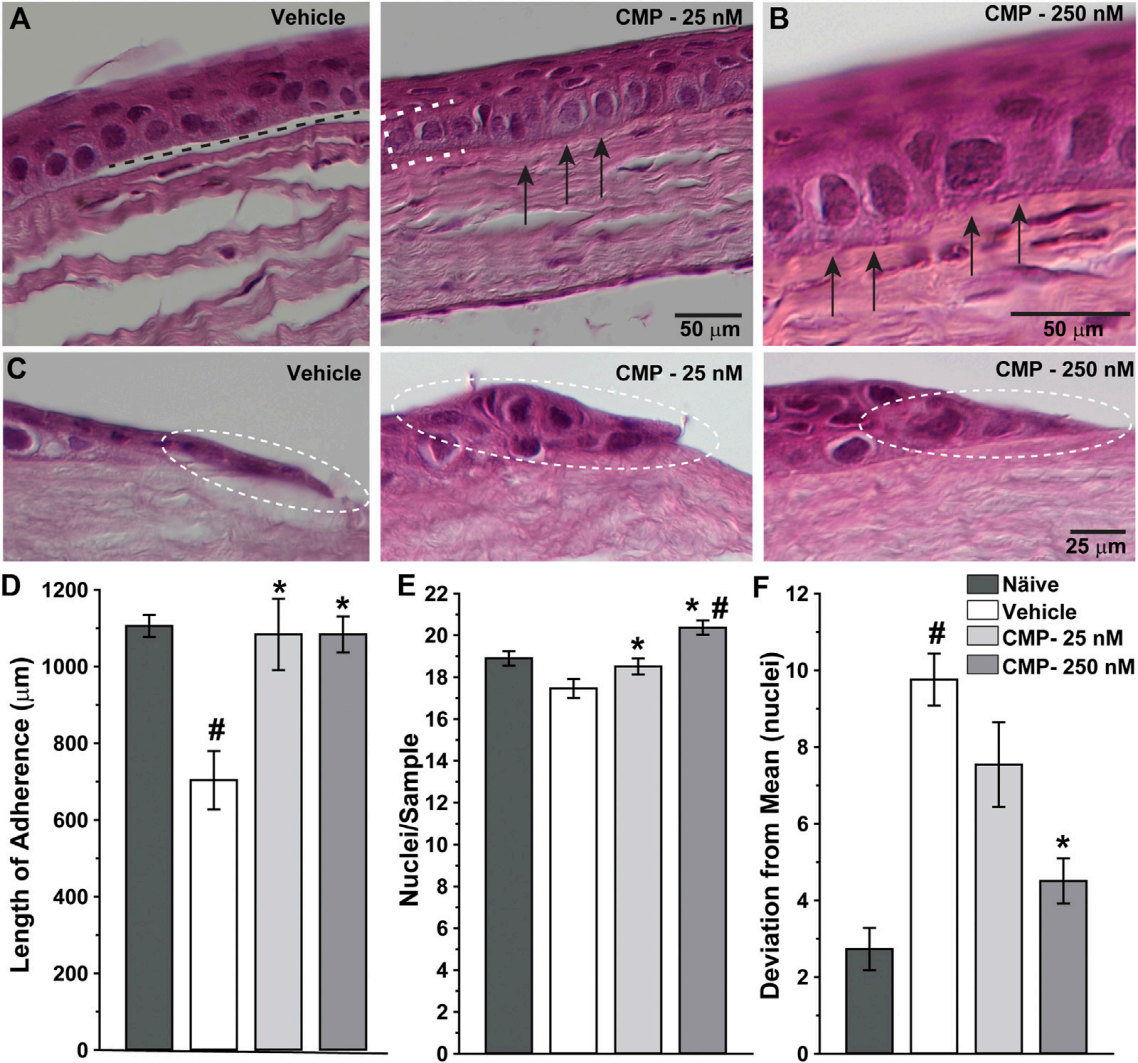
CMP enhances the structure of healing corneal epithelium. Representative differential interference contrast images of histological sections with hematoxylin and eosin staining through regenerated epithelium (**A**, **B**) and the proliferative edge **(C)** 24 h following induced corneal injury. While vehicle-treated eyes demonstrated frequent gaps beneath the epithelium (**A**, left; dashed line), treatment with CMP enhanced adherence of the basal layer (brackets) to the underlying anterior stromal surface (arrows) for both 25 (**A**, right) and 250 nM (**B**, at higher magnification) concentrations of CMP. At the proliferative edge of the wound **(C)**, compared to a vehicle, CMP increases the number of new epithelial cells adhering to the corneal stroma (circles). **(D)** Length of contiguous segments of adherence between basal epithelium and underlying stromal layer calculated from random samples of histological sections through cornea 24 h following epithelial removal (*n* = 6–11 each). Compared to naïve eyes, vehicle-treated corneas demonstrated a 36% reduction in the average length of adherent surface (#: *p* < 0.001). Treatment with CMP significantly increased adherence compared to a vehicle (*: *p* ≤ 0.02), comparable to adherence in naïve corneas (*p* ≥ 0.37). **(E)** The number of epithelial cell nuclei in basal layer quantified in random samples (*n* = 29–100 each). Treatment with CMP increased the number of cells per sample significantly compared to a vehicle (*: *p* ≤ 0.04); the number of cells following 250 nM CMP treatment exceeded naïve (#: *p* = 0.008) **(F)**. Deviation in the number of epithelial nuclei in each random sample from (**A**) compared to the mean number for each cohort. The vehicle group was significantly more variable than naïve (#: *p* < 0.001); treatment with 250 nM CMP reduced deviation by 54% compared to a vehicle (*: *p* < 0.001) but remained higher than naïve (*p* = 0.04).

Next, we determined how treatment with CMP influenced corneal healing at the structural level by examining histological sections through the wound region. High magnification images revealed significant histological differences between vehicle- and CMP-treated eyes (Figure 4). Vehicle-treated corneas demonstrated intermittent gaps between the basal epithelium and underlying stromal surface (Figure 4A, left), while CMP-treated eyes demonstrated a higher degree of adherence (Figure 4B). This was so for both concentrations of CMP tested (Figures 4A, B, right). At the proliferative edge of the epithelium, the basal layer in vehicle-treated eyes appeared thinner and less organized than that in the CMP-treated ones (Figure 4C).

When quantified, the total length of adherence between the basal epithelium and underlying substrate decreased after wounding by 36% in vehicle-treated eyes compared to naïve corneas prepared and sectioned in the same way (Figure 4D). In contrast, treatment with CMP at both concentrations increased adherence to the same levels as naïve (*p* ≥ 0.37). Similarly, the number of epithelial cells per unit area in the basal epithelial layer was greater for both CMP concentrations compared to a vehicle (*p* ≤ 0.04) and was higher than naïve for 250 nM (*p* = 0.008; Figure 4E). Finally, we determined whether CMP influenced variability in the organization of the epithelial layer. We quantified the number of nuclei in adjacent segments of a fixed area for each cornea and measured how the number in each segment deviated from the average for the entire sample. By this measure (Figure 4F), vehicle-treated corneas demonstrated three-fold greater variability than naïve (*p* < 0.001). Treatment with 250 nM CMP reduced deviation by 54% compared to a vehicle (*p* < 0.001) but remained higher than naïve (*p* = 0.04). Treatment with 25 nM CMP improved variability but not significantly compared to a vehicle (*p* = 0.10). Thus, CMP improved regeneration and organization of the basal epithelium with greater adherence of the epithelial cells to the underlying stromal surface.

## Discussion

The cornea is a highly collagenous structure susceptible to environmental and other insults and a number of diseases affecting the ocular surface (Eghrari et al., 2015). Our results show that CMPs have the potential as therapeutic agents to address conditions that challenge the cellular integrity of the cornea, albeit an effective treatment must demonstrate long-term benefits to vision without compromising corneal transparency. This CMP by design bypasses normal, intact collagen but anneals to and directly repairs damaged collagen (Chattopadhyay et al., 2012; Chattopadhyay et al., 2016; Chattopadhyay and Raines, 2014). Our results suggest that treatment with CMP improved the regularity of collagen *in vitro*, as shown by increased parallelism of apparent type I collagen strands damaged by collagenase and treated with CMP vs. a vehicle (Figure 1). While this is an indirect measure, the pattern is similar to the aligned orientation of individual collagen strands reported in previous *in vitro* studies (Hapach, et al., 2015; Kivanany et al., 2018).

Treatment with CMP also had beneficial effects *in vivo*. Topical application accelerated healing following an acute injury to the mouse eye that removed the corneal epithelium from some 70% of the corneal surface (Figure 2). A multivariable analysis of variance indicated a significant difference in the ratio of residual to initial wound size over time post-injury between CMP and a vehicle (Figure 3A). Clustering of the ratio at lower values for both 16 and 24 h suggests that CMP treatment induced a faster rate of healing, especially between 16 and 24 h (Figure 3B). CMP treatment also significantly enhanced adherence of the basal epithelial cell layer to the underlying substrate and resulted in a greater number of epithelial cells per unit area (Figure 4), including the proliferative edge of the epithelium where renewal occurs (Ljubimov and Saghizadeh, 2015; Mobaraki et al., 2019). Collagen peptides derived from fish scales similarly enhance adherence of the corneal epithelium and reduce inflammatory signaling (Subhan et al., 2017). Our CMP also increased the regularity of the epithelium, with less variability in the number of cells between adjacent samples compared to a vehicle (Figure 4F).

In total, our results indicate that CMP accelerates healing, presumably through its known action of intercalating into damaged collagen strands Chattopadhyay et al. (2012), presumably where the epithelial basement membrane was removed and stroma disturbed in our wound model. In most species, the collagenous substrate between the basement membrane and stroma corresponds to Bowman’s layer, which is underdeveloped in mice (Wilson, 2020). Even so, the specificity of our CMP to intercalate exclusively in damaged collagen strands suggests potential utility in clinical conditions that damage the collagenous complex consisting of the basement membrane, Bowman’s layer, and the stroma. In terms of the direct action of CMPs, prior work using similar CMPs demonstrates unequivocally their exclusive intercalation into and healing of fragmented collagen helices; this design underlies their efficacy in other wound models (Chattopadhyay et al., 2012; Chattopadhyay et al., 2016; Chattopadhyay and Raines, 2014). However, this action does not preclude the possibility that by intercalation, they occlude enzymic active sites, thereby reducing further degradation. Single strand forms of CMPs selectively target degraded regions of a collagen triple-helix and strongly hybridize to type I collagen fibrils (Wang et al., 2005; Li and Yu, 2013). An intact and well-organized collagen scaffold is known to be a prerequisite for regrowth of the basal epithelium, the only mitotic cells in the layer (Ljubimov and Saghizadeh, 2015). The basal epithelial cells create a basement membrane enriched in type IV collagen as they migrate from the corneal edge, a process that is challenged in many diseases and conditions (Torricelli et al., 2013). Type IV collagen, like all non-fibrillar collagen types, contains a type I domain which is a site of collagenase damage (Exposito et al., 2010). The CMP could act directly at that site through its propensity for binding to damaged type I collagen (Dones et al., 2019). Both superficial underlying stromal tissue and mid-stroma contain primarily type I collagen (Ihanamäki et al., 2004; Meek, 2009; Wilson, 2020). Thus, alternatively, or in a combined action, the CMP could repair the distressed apical surface of the stroma and, as such, providing an enhanced substrate for cell migration and ultimate restoration of homeostasis. Healthy triple helix collagen is known to be involved in promoting cellular signaling that is a hallmark of normal tissue homeostasis (San Antonio et al., 2020). Thus, CMPs could contribute to the rate and quality of corneal healing through the restoration of ligand binding that is involved in cellular signaling in healthy tissue, perhaps thereby also providing a potential secondary anti-inflammatory effect (Lebbink et al., 2006; An et al., 2016).

Similar CMPs have demonstrated efficacy not only in detecting damaged collagen Takita et al. (2018), Dones et al. (2019) but also in promoting healing of skin wounds (Chattopadhyay et al., 2016). Synthesized collagen peptides that promote tear adherence to the ocular surface also facilitate epithelium stabilization in animal models of dry eye disease (Lee et al., 2017). There are myriad clinical implications of having a therapeutic tool available to directly repair damaged collagen in ocular surface disease, especially those affecting corneal collagen (Baratta et al., 2021). These include dry eye syndrome, keratitis, corneal ulceration, laceration and melting, and recurrent corneal erosion. There are few therapeutic options for these indications, and those that are available are relatively slow acting and lack effectiveness at directly healing the structural backbone of the cornea. CMPs offer an additional potential therapeutic approach for treating a wide variety of ophthalmic indications where collagen damage is present. The potential ability of CMPs to reduce inflammation and scar formation, which can represent abnormal upregulation of collagen repair, also would be an important area for further research.

## Data Availability

The original contributions presented in the study are included in the article/Supplementary Material. Further inquiries can be directed to the corresponding author.
